# Life‐history attributes of Arctic‐breeding birds drive uneven responses to environmental variability across different phases of the reproductive cycle

**DOI:** 10.1002/ece3.8448

**Published:** 2021-12-13

**Authors:** Daniel R. Ruthrauff, Vijay P. Patil, Jerry W. Hupp, David H. Ward

**Affiliations:** ^1^ U.S. Geological Survey, Alaska Science Center Anchorage Alaska USA

**Keywords:** Arctic, environmental variability, life history, nutrient storage strategies, phenology, reproduction

## Abstract

Animals exhibit varied life‐history traits that reflect adaptive responses to their environments. For Arctic‐breeding birds, traits related to diet, egg nutrient allocation, clutch size, and chick growth are predicted to be under increasing selection pressure due to rapid climate change and increasing environmental variability across high‐latitude regions. We compared four migratory birds (black brant [*Branta bernicla nigricans*], lesser snow geese [*Chen caerulescens caerulescens*], semipalmated sandpipers [*Calidris pusilla*], and Lapland longspurs [*Calcarius lapponicus*]) with varied life histories at an Arctic site in Alaska, USA, to understand how life‐history traits help moderate environmental variability across different phases of the reproductive cycle. We monitored aspects of reproductive performance related to the timing of breeding, reproductive investment, and chick growth from 2011 to 2018. In response to early snowmelt and warm temperatures, semipalmated sandpipers advanced their site arrival and bred in higher numbers, while brant and snow geese increased clutch sizes; all four species advanced their nest initiation dates. During chick rearing, longspur nestlings were relatively resilient to environmental variation, whereas warmer temperatures increased the growth rates of sandpiper chicks but reduced growth rates of snow goose goslings. These responses generally aligned with traits along the capital‐income spectrum of nutrient acquisition and altricial–precocial modes of chick growth. Under a warming climate, the ability to mobilize endogenous reserves likely provides geese with relative flexibility to adjust the timing of breeding and the size of clutches. Higher temperatures, however, may negatively affect the quality of herbaceous foods and slow gosling growth. Species may possess traits that are beneficial during one phase of the reproductive cycle and others that may be detrimental at another phase, uneven responses that may be amplified with future climate warming. These results underscore the need to consider multiple phases of the reproductive cycle when assessing the effects of environmental variability on Arctic‐breeding birds.

## Graphical abstract

Arctic‐breeding birds exhibit a variety of life‐history traits that serve to maximize reproductive output, but certain traits may vary in their efficacy under variable environmental conditions. We compared four species of migratory birds breeding at a site in Arctic Alaska, USA, and determined that certain life‐history traits of black brant and snow geese are especially beneficial with respect to the timing of and investment in breeding, but that gosling growth in these species may be negatively affected under warming climates. In contrast, chick growth of semipalmated sandpipers was more rapid in warm conditions irrespective of food abundance, while rates of Lapland longspur chick growth were relatively invariable with respect to environmental conditions.

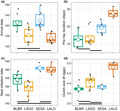

## 1 INTRODUCTION

Compared to temperate and tropical ecosystems, the reproductive period of most animals in the Arctic is compressed due to the brief availability of food and short period of suitable weather (MacLean & Pitelka, 1971; Wingfield & Hunt, 2002). Nevertheless, the sheer abundance of resources at these sites supports the reproduction of a diversity of animal groups that employ a variety of life‐history strategies to exploit conditions in the Arctic. For migratory birds, these traits include reproductive strategies along the capital‐income spectrum of resource allocation (Drent & Daan, 1980; Klaassen et al., 2001), the altricial–precocial spectrum of chick development (Starck & Ricklefs, 1998), and variable investments in clutch size (Jetz et al., 2008; Winkler & Walters, 1983). Life‐history theory predicts an optimization of such traits within a species (Roff, 2002; Stearns, 1992), and comparison of these traits among co‐occurring species over multiple breeding occasions provides insight into factors that promote successful reproduction across varying environmental conditions.

Such comparisons are especially relevant in the Arctic considering the rapid pace of ecosystem change due to climate effects (Berteaux et al., 2004; Hoffmann & Sgrò, 2011). The effects of climate change are disproportionately expressed at high‐latitude regions (Arctic Climate Impact Assessment, 2004; IPCC, 2021), where the rate of warming is rapid (Bekryaev et al., 2010; Serreze & Barry, 2011), the onset of spring is advancing (Høye et al., 2007; Parmesan & Yohe, 2003), and the growing season is lengthening (Piao et al., 2007; Tucker et al., 2001). In addition to these steady trends, the Arctic is also experiencing an increased frequency of punctuated, extreme climatic events (Landrum & Holland, 2020). Taken together, these rapidly changing conditions present new challenges to organisms already inhabiting extreme Arctic environments (Berteaux et al., 2004; Gilg et al., 2012).

We studied the breeding ecology of migratory birds at an Arctic site in Alaska to assess the response of the avian community to climate‐related environmental variation. We focused our research efforts on the four most common species at the site: two geese (black brant [*Branta bernicla nigricans*] and lesser snow geese [*Chen caerulescens caerulescens*]), one shorebird (semipalmated sandpiper [*Calidris pusilla*]), and one passerine (Lapland longspur [*Calcarius lapponicus*]). These species encompass a range of life‐history traits relating to reproductive effort (Table 1). Black brant (hereafter brant) and lesser snow geese (hereafter snow geese) are large‐bodied, herbivorous waterfowl. Both species deposit endogenous nutrients into eggs (Schmutz et al., 2006; Sharp et al., 2013), but snow geese acquire relatively more exogenous nutrients from Arctic plants when foraging conditions prior to nesting are favorable (Hupp et al., 2018). In contrast, semipalmated sandpipers and Lapland longspurs (hereafter longspurs) are small‐bodied birds that rely on exogenously derived nutrients (insects and seeds) for egg production (Hobson & Jehl, 2010; Klaassen et al., 2001; Meijer & Drent, 1999). Brant (2–6 eggs; Lewis et al., 2020), snow geese (2–6 eggs; Hamann et al., 1986), and longspurs (2–8 eggs; Custer & Pitelka, 1977) also regulate their reproductive investment by producing variable numbers of eggs, but the clutch size of semipalmated sandpipers is essentially invariant (4 eggs; MacLean, 1972; Sandercock, 1997). Finally, brant (Lewis et al., 2020), snow geese (Mowbray et al., 2020), and semipalmated sandpipers (Holmes & Pitelka, 1968) produce precocial chicks that exit the nest shortly after hatch and are self‐feeding, but longspur nestlings are altricial and derive all their food resources from the provisioning efforts of adult longspurs (Custer & Pitelka, 1977).

**TABLE 1:** Life‐history variation among reproductive traits for four species of Arctic‐breeding bird, black brant (BLBR), lesser snow goose (LSGO), semipalmated sandpiper (SESA), and Lapland longspur (LALO)

Life‐history trait	Species			
	BLBR	LSGO	SESA	LALO
Nutrient source	Herbaceous	Herbaceous	Insect	Insect, seed
Resource allocation	Endogenous	Flexible	Exogenous	Exogenous
Clutch investment	Variable; 2–6 eggs	Variable; 2–6 eggs	Invariant; 4 eggs	Variable; 2–8 eggs
Chick growth	Precocial	Precocial	Precocial	Altricial

The interspecific variation in life‐history traits represents functional attributes (e.g., resource use and allocation, reproductive investment, modes of chick growth) that reflect selective forces acting under a rapidly changing climate (Berteaux et al., 2004; Gienapp et al., 2008). To this end, we monitored the annual arrival, pre‐lay duration, nest initiation, clutch size, and nesting effort of the four study species and measured relevant environmental variables across the breeding season. We predicted that geese would exhibit more flexibility to interannual variability in spring temperature and snowmelt compared to semipalmated sandpipers and longspurs due to their comparatively greater endogenous reserves and relative flexibility along the capital‐income spectrum. We reasoned that geese would exhibit flexibility by adjusting the timing of their breeding and reproductive investments more than semipalmated sandpipers and longspurs.

Species may possess traits that promote resilience during one phase of the reproductive cycle but have other traits which may be detrimental at other phases. As such, assessing the response of species to shared environmental conditions across different phases of the reproductive cycle (Nolet et al., 2020) provides more meaningful insights into reproductive outcomes than assessments focused on just one phase. In addition to monitoring responses during the prebreeding and nesting periods, we also monitored the growth of chicks of these four species in conjunction with climatic variables and measures of the seasonal availability of food resources. Because numerous studies have demonstrated that rapid growth of avian young yields larger chicks (Larsson & Forslund, 1991; Ruthrauff & McCaffery, 2005) that survive at higher rates (Lindholm et al., 1994; Naef‐Daenzer et al., 2001) and have a higher probability of recruitment (Cooch et al., 1993; Magrath, 1991; Sedinger et al., 1995) than smaller same‐age chicks, variation in chick growth reflects a meaningful demographic response to environmental variability. We predicted that the young of brant, snow geese, and semipalmated sandpipers would be more sensitive to variation in food abundance due to their precocial nature. We predicted that this would be reflected by relatively strong variation in body mass as a function of food abundance compared to longspur nestlings, which are provisioned entirely by adults. Taken together, assessments of climatological (temperature, wind, and snow cover) and environmental (seasonal availability and absolute abundance of food resources) factors in relation to life‐history traits across multiple phases of the reproductive cycle elucidate characteristics of Arctic‐breeding birds that may mitigate negative effects of future climate change.

## 2 MATERIALS AND METHODS

We conducted our study on the Arctic Coastal Plain of Alaska at the Colville River Delta (70.44°N, 150.67°W). This site is ~5 km from the Beaufort Sea and is a lowland ecosystem of lakes, polygonal ponds, graminoid‐dominated wetlands, dune ridges, and upland tundra communities (Kessel & Cade, 1958; Walker, 1983). Our study period was from late May to late July 2011–2018, but not all data were collected in all years because we added new aspects to the study over time. We conducted systematic searches for semipalmated sandpiper (2011–2018) and Lapland longspur (2015–2018) nests across a 2.6‐km^2^ plot adjacent to our camp, and we traveled by boat within 15 km of our camp to monitor goose nests at nearby colonies. To standardize search efforts across known‐area plots for geese, from 2015 to 2018, we counted the number of brant and snow goose nests at 61 randomly selected circular plots (15‐ or 25‐m radius, depending on nest density) within these nesting areas. We monitored nests of semipalmated sandpipers and longspurs discovered on our core study plot, and brant and snow geese at their colonies. We calculated the average clutch size for each species for each year of study from these samples. To evaluate nesting effort relative to environmental variables from 2015 to 2018, we counted nests of shorebirds and longspurs in our core study plots and goose nests in random plots.

If a nest was found during laying, we estimated initiation date by back‐dating by the number of eggs found at discovery based on published estimates of egg‐laying rates (Alisauskas & Ankney, 1992; Hussell & Montgomerie, 2020; Sandercock, 1998). For nests found with a complete clutch, we used an egg candling technique (Weller, 1956) for brant and snow geese, and an egg floatation technique (Liebezeit et al., 2007) for semipalmated sandpipers to estimate embryo age and back‐date accordingly. For longspur nests that were found with complete clutches, we were only able to estimate initiation dates for nests that subsequently hatched, wherein we back‐dated from the hatch date based on the nest's clutch size and application of a 12‐day incubation period (Hussell & Montgomerie, 2020).

### 2.1 Reproductive and environmental phenology

We maintained daily checklists to determine the first arrival date for each species at our study site. If a species was present at our site upon our arrival, we instead used the first‐arrival information collected from a site 10 km from our camp (see Ward et al., 2016). Once nesting commenced, we determined the initiation date of each nest using standard techniques (e.g., egg floatation, egg candling, back‐calculating from hatch). Nests of longspurs were only monitored from 2015 to 2018, but we monitored nests of the other three species from 2011 to 2018.

We collected a suite of environmental variables at or near our study site. We recorded the percent cover of snow at 10 (2018) or 20 (2011–2017) 25‐m radius plots that we monitored each year. We assessed snow cover upon arrival at the field site and every 2–6 days (typically 2 days) thereafter until snow cover averaged <5%. We averaged the daily values of snow cover across the plots and used the annual date when snow cover averaged 50% as an indicator of annual snowmelt. To characterize spring temperatures that preceded our arrival at the field site, we accessed weather observations at a site 10 km away (Colville Village, Alaska; National Oceanic & Atmospheric Administration, 2020) and determined values for accumulated thaw‐degree days for each year. We also used an on‐site weather station to record hourly temperature and wind speed, values which we summarized in running 3‐day averages for use in chick‐growth analyses.

### 2.2 Resource abundance

We monitored the seasonal abundance of the primary food resources available to juvenile birds at our site. We began monitoring both herbivore and insectivore food resources as early in the season as possible based on snow cover and ground thaw and monitored these resources throughout the period of chick growth. For brant and snow geese, this involved estimating the biomass of the halophytic sedge *Carex subspathacea* (Gadallah & Jefferies, 1995a; Hupp et al., 2017; hereafter *subspathacea*) by measuring the Normalized Difference Vegetation Index (NDVI). We used a handheld spectrometer (PP Systems, Inc.) and calculated NDVI based on WorldView‐2 spectral band values (Hogrefe et al., 2017). We measured NDVI across 6–10 sampling occasions per year from 2012 to 2017 at 5–8 1.5 × 1.7 m^2^ plots that were grazed by geese. We estimated seasonal trends in the availability of *subspathacea* (g/m^2^) using analytical procedures and models derived at our study site and described in detail by Hogrefe et al. (2017). We fit lognormal models to each year's sample averages to describe the nonlinear seasonal trends in biomass and used daily predictions from these year‐specific estimates to determine the biomass of *subspathacea* that was available to goslings when they were 15 days old.

Chicks of semipalmated sandpipers (Holmes & Pitelka, 1968) and longspurs (Custer et al., 1986) are insectivores. To monitor the availability of these food resources, we collected surface‐active arthropods every three days from ten modified Malaise traps following protocols of the Arctic Shorebird Demographic Network (Brown et al., 2014; Saalfeld et al., 2019). We collected arthropod samples at three‐day intervals from 2015 to 2017 and stored samples in ethanol for enumeration and identification to family or order (as practical) after the field season. We applied established length–mass relationships to estimate the biomass (mg) of prey items (see Saalfeld et al. [2019] for details). Chicks of sandpipers (Holmes & Pitelka, 1968) and longspurs (Custer & Pitelka, 1978) consume a diversity of arthropod prey, but cannot consume large bees in the order Hymenoptera due to gape restrictions. As such, we removed large bees from our samples, but otherwise summed biomass values across taxonomic groups for each trap during each collection period and averaged these values across all ten traps to determine the average arthropod biomass per 3‐day collection period.

### 2.3 Chick growth

To monitor the growth of brant and snow goose goslings, we marked the webbing of one foot of a sample of goslings of both species with uniquely numbered tags (Alliston, 1975) from 2012 to 2017. Goslings were marked either as they hatched or shortly after they hatched, and to minimize disturbance within breeding colonies, we did not weigh the goslings when we applied the web tags. Goslings were recaptured once and weighed along with tending adults in banding drives conducted in late July and early August (Hupp et al., 2017). Goslings of both species were sexed via cloacal eversion during the banding process (Hanson, 1967).

We collected repeat measures of body mass from hatch until fledge on chicks of semipalmated sandpipers and longspurs. For semipalmated sandpipers, we visited nests at hatch and banded the chicks with uniquely numbered U.S. Geological Survey metal bands. Chicks departed the nest shortly after hatch, and we attempted to recapture chicks at 5‐day intervals, but also collected mass measurements when we opportunistically encountered broods. Longspur chicks are too small to retain leg bands at hatch, so we did not uniquely mark longspur chicks. We visited longspur nests and weighed nestlings as soon as possible after the first egg in each nest hatched and weighed nestlings at approximately 3‐day intervals thereafter. We monitored the growth of semipalmated sandpiper chicks and longspur nestlings from 2015 to 2017. For both of these species, the sex of the chicks was unknown. We weighed the chicks of all four focal species with electronic balances accurate to ±0.1 g for semipalmated sandpipers and longspurs, and ±5 g for geese.

### 2.4 Analysis

#### 2.4.1 Reproductive phenology and investment

We compared similar environmental metrics across groups in our analyses and modified as necessary due to inherent differences in relevant life‐history traits. Specifically, we first compared the arrival date, duration of the pre‐lay period (the number of days between when a species was first detected at our study site and that species’ mean date of nest initiation), mean date of nest initiation, and mean clutch size of the four species using standard ANOVA techniques and conducted Tukey's HSD tests for post hoc examination of differences between species. Next, to assess evidence for species‐specific responses to snow cover (date of 50% snow cover each year) and temperature (accumulated thaw‐degree days from 1 January–10 June each year; Table 2a), we estimated slope parameters of least‐squares linear regression models. We fit models with unique slopes for each species (i.e., models with interactions between species and environmental variables), with arrival date, duration of the pre‐lay period, mean date of nest initiation, mean clutch size, and nesting effort (the annual number of nests of each species enumerated on known‐area plots) as response variables. For these assessments, we considered results to be biologically meaningful at α = .05.

**TABLE 2:** Predictor variables used to assess variation in (a) reproductive phenology and investment and (b) chick growth of black brant (BLBR), lesser snow goose (LSGO), semipalmated sandpiper (SESA), and Lapland longspur (LALO) breeding at the Colville River, Alaska, 2011–2018

Variable	Species			
	BLBR	LSGO	SESA	LALO
(a) Reproductive phenology and investment				
Snow cover	Annual date 50% snow cover			
Temperature	Annual thaw‐degree days (TDD) from 1 Jan–10 Jun			
(b) Chick growth				
Resource abundance	*Carex subspathacea* biomass (g/m^2^) on day 15		Ave. mg arthropods 3‐day^−1^	
Nest timing	Incubation – date 50% snow		Initiation – date 50% snow	
Temperature	Lifetime TDD (°C)		3‐day ave. (°C)	
Wind	3‐day ave. (m/s)			

#### 2.4.2 Chick growth

We compared similar environmental metrics across all four chick‐growth model sets (Table 2b). We determined the influence of absolute resource abundance (biomass of *subspathacea* [g/m^2^] and arthropod biomass [mg per trapping period] for herbivores and insectivores, respectively) on growth in ways that reflected relevant life‐history traits of each species. Due to digestive constraints, goslings are sensitive to variations in the abundance and quality of their forage resources (Gadallah & Jefferies, 1995b; Sedinger & Raveling, 1988), constraints believed to be especially pronounced during periods of rapid growth (Lepage et al., 1998). As such, we used annual, season‐long estimates of daily *subspathacea* biomass to determine the biomass available to each web‐tagged gosling when they were 15 days old, an age overlapping the period of most‐rapid growth in both species of geese (Ankney, 1980; Sedinger & Flint, 1991). In contrast, chicks of semipalmated sandpipers and longspurs are small‐bodied and respond quickly to short‐term fluctuations in arthropod biomass (Kwon et al., 2019; Schekkerman et al., 2003). Thus, for insectivores, we associated chick‐mass measurements to the arthropod biomass sample that was collected most closely in time across the 3‐day, season‐long arthropod sampling interval. To assess potential effects of the relative timing of nesting with respect to annually variable spring environmental conditions, we calculated the difference between each nests’ date of initiation (insectivores) or onset of incubation (herbivores) and year‐specific dates of 50% snow cover. Finally, we assessed the effects of temperature and wind on chick growth. Similar to how we quantified effects of food biomass, we reasoned that larger bodied herbivore chicks were more resilient to short‐term changes in temperatures than small‐bodied insectivores. Thus, we characterized temperatures for each gosling by summing thaw‐degree values from hatch until capture. For the smaller bodied chicks of semipalmated sandpipers and longspurs, we used short‐term temperature summaries to represent the effects of temperature on chick growth by applying 3‐day running averages of temperature (°C) across the season and associated each mass measurement with the corresponding 3‐day average value. We similarly calculated 3‐day running averages of wind speed (m/s) and linked these values with body mass measurements for chicks of all four species.

To assess factors that influenced the growth of brant and snow goose goslings, we fit linear models to estimate body mass as a function of gosling age and sex. Because we weighed goslings only once, we did not estimate a growth curve for the period from hatch until fledge, but instead modeled the growth of both goose species over the span of ages represented in our samples (see Hupp et al., 2017). Although the mass gain of goslings from both species is nonlinear overall (Ankney, 1980; Sedinger & Flint, 1991), gosling growth rates are well approximated by a linear fit for the period shortly prior to fledging over which we recaptured goslings (Cooch et al., 1991). For semipalmated sandpiper chicks and longspur nestlings, we log_10_‐transformed both mass and age to reduce inherent patterns of unequal variance from hatch until fledging (NB: goslings were weighed only during the linear phase of their growth cycle such that variances were equal across ages, making log transformation unnecessary). Because we uniquely marked goslings and semipalmated sandpiper chicks, we modeled the growth of individuals of these species, but used the average brood mass per nest visit as our response variable for longspurs. Finally, to focus on posthatch factors affecting chick growth, we excluded mass measurements collected on the day of hatch for longspurs. For semipalmated sandpipers, we similarly censored values collected prior to two days of age because chicks of Arctic‐breeding shorebirds rely primarily on internal yolk reserves to fuel growth after hatch (Norton, 1973; Schekkerman et al., 1998).

Chick‐growth comparisons among our study species represent our best efforts to balance biological reality with analytical necessity. For instance, exploratory modeling efforts involving nonlinear mixed‐effects models (see Tjørve & Tjørve, 2010) were unavoidably complex and failed to converge. Other approaches that used residuals from best‐fit, nonlinear growth models as predictor variables in a mixed‐effects framework (see Saalfeld et al., 2019) had relatively low marginal and conditional *R* ^2^ values (Nakagawa & Schielzeth, 2013; see further). Ultimately, we fit linear‐mixed effects models using least‐squares regression to each species independently using the package “lme4” (Bates et al., 2015), an approach that satisfied underlying model assumptions and yielded improved measures of objective model performance (Nakagawa & Schielzeth, 2013). We standardized all environmental variables to have a mean of 0 and standard deviation of 1 to facilitate model interpretation. We fit an intercept‐only (null) model in all model sets, and otherwise included chick age (semipalmated sandpipers and longspurs) or gosling age and sex (brant and snow geese) as unstandardized covariates in all models. We combined these covariates along with additive combinations of the aforementioned environmental variables to create an all‐subsets model set of 17 models for each species. We fit mixed‐effects models in order to control for observations of multiple individuals from the same nest (all species) and repeat measures of individual semipalmated sandpiper chicks (Zuur et al., 2009). We employed multimodel comparisons to rank the support of each model based on Akaike's information criterion adjusted for small sample size (AIC*_c_*) and averaged model results for each species in proportion to Akaike weights *w_i_* following the approaches of Burnham and Anderson (2002). We calculated the conditional and marginal *R* ^2^ of each model using the R package “piecewiseSEM” (Lefcheck, 2016) to assess objective model performance (Nakagawa & Schielzeth, 2013) and performed multimodel comparisons and model averaging using the R package “AICcmodavg” (Mazerolle, 2019). We considered predictor variables with 85% confidence intervals that did not overlap zero (Arnold, 2010) to be biologically meaningful and generated model‐averaged predictions for each species using contrasting values of these biologically meaningful predictor variables to visualize chick mass under varying conditions. Specifically, we generated predictions representing growth under what we term optimal (i.e., 75th‐quartile values for predictor variables with positive parameter estimates, 25th‐quartile values for predictor variables with negative parameter estimates) and suboptimal (i.e., 25th‐quartile values for positive parameters, 75th‐quartile values for negative parameters) conditions. For environmental variables whose model‐averaged parameter estimates overlapped zero (i.e., uninformative predictors), we used the mean observed value when generating growth predictions. All analyses were performed in R (R Core Team, 2021), and values represent mean ± SD unless otherwise noted.

## 3 RESULTS

Over the course of our study, we monitored 1447 brant nests, 1374 snow goose nests, 821 semipalmated sandpiper nests, and 142 longspur nests. Our study area experienced highly variable environmental conditions. In spring, the date of 50% snow cover ranged over a nearly three‐week period from 20 May (2016)–8 June (2013) and values for accumulated thaw‐degree days 1 January–10 June ranged from 7.2 (2018)–56.1 (2015). Warmer springs had earlier dates of snowmelt (adjusted *R* ^2^ = .53, *p* = .02), but our study period also encompassed years with mixed patterns between the two variables (e.g., years with relatively early [2015] or late [2013] snowmelt that did not reflect prevailing spring temperature). We also recorded prolonged periods of high (e.g., average temperature 17.3°C from 13–16 July 2016) and near‐freezing (e.g., average temperature 3.4°C from 7–13 July 2015) temperatures during periods of chick growth.

### 3.1 Variation in reproductive phenology and investment

**FIGURE 1:**
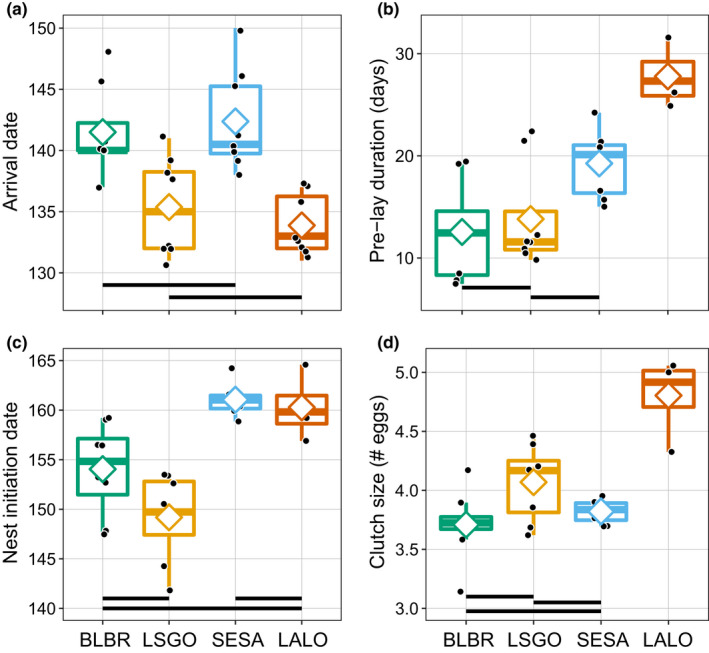
Variation in arrival date (a), pre‐lay interval (b), date of nest initiation (c), and clutch size (d) and of black brant (BLBR), snow geese (LSGO), semipalmated sandpipers (SESA), and Lapland longspurs (LALO) at a site on the Colville River, Alaska, 2011–2018. Horizontal lines represent the median, diamonds the mean, black circles the actual annual values, boxes the 25^th^ and 75^th^ percentiles, and whiskers the range of values. All interspecific comparisons of these values were statistically significant (*p* < .05) based on post hoc Tukey HSD comparisons, except for comparisons linked by horizontal bars at bottom of each plot (e.g., mean arrival dates of black brant and semipalmated sandpipers do not statistically differ; (a). For (a) and (c), ordinal date 140 is 20 May

Mean dates of arrival at the breeding site (*F*_3,28_ = 11.2, *p* < .001; Figure 1a), duration of the pre‐lay period (*F*_3,24_ = 13.7, *p* < .001; Figure 1b), nest initiation (*F*_3,24_ = 16.9, *p* < .001; Figure 1c), and clutch size (*F*_3,24_ = 17.0, *p* < .001; Figure 1d) varied considerably among the four species. Regardless of spring conditions, snow geese (15 May ± 4.0 days) and longspurs (14 May ± 2.4 days) arrived earliest each spring (Figure 1a), followed approximately one week later by brant and semipalmated sandpipers (both 22 May ± 3.6 days and ±4.2 days, respectively). Early arrival did not necessarily confer rapid nesting, however, the pre‐lay period (Figure 1b) was longest for longspurs (mean 27.8 ± 2.9 days), followed by semipalmated sandpipers (19.3 ± 3.2 days), snow geese (13.8 ± 5.1 days), and brant (12.6 ± 4.7 days). Due to their early arrival and short pre‐lay period, snow geese consistently initiated nests before the other three species (Figure 1c). Mean nest initiation was 30 May (±4.6 days) for snow geese, 4 June (±5.2 days) for brant, 8 June for longspurs (±9.3 days), and 10 June (±5.4 days) for semipalmated sandpipers. Longspurs had the largest clutches (4.8 ± 1.1 eggs; Figure 1d), followed by snow geese (4.0 ± 1.2 eggs), semipalmated sandpipers (3.8 ± 0.5 eggs), and brant (3.7 ± 1.0 eggs).

**FIGURE 2:**
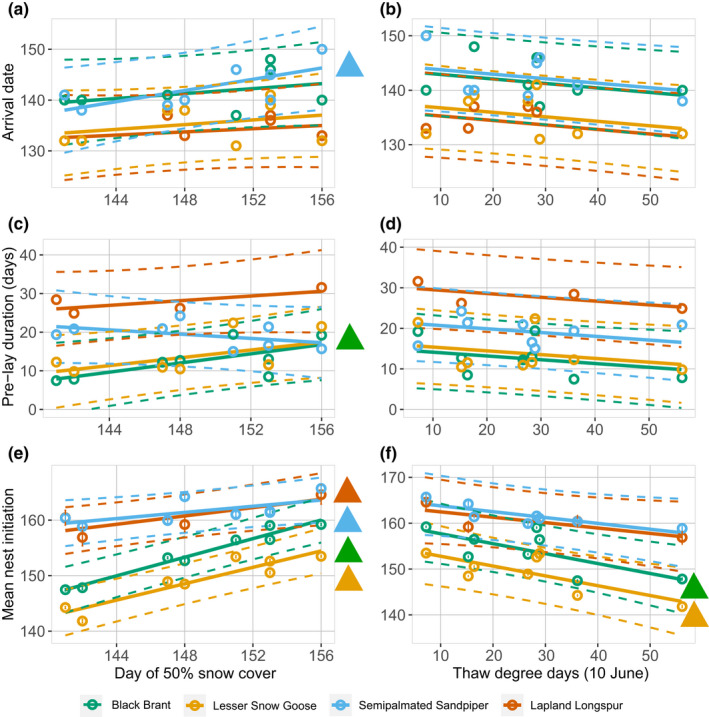
Effect of interannual differences in the date of 50% snow cover (left column) and accumulated thaw‐degree days on through 10 June (right column) on the arrival date (a and b), pre‐lay interval (c and d), and mean date of nest initiation (e and f) of black brant, snow geese, semipalmated sandpipers, and Lapland longspurs at a site on the Colville River, Alaska, 2011–2018. Circles represent year‐specific values (±SE for nest initiation), and solid lines represent the best‐fit least‐squares regression ±95% prediction interval (dashed lines). Species‐specific colored triangles to right of figures represent slope parameters that significantly differ from zero. See Table 3 for estimates (±95% confidence interval) of slope parameters. Ordinal date 148 is 28 May

**FIGURE 3:**
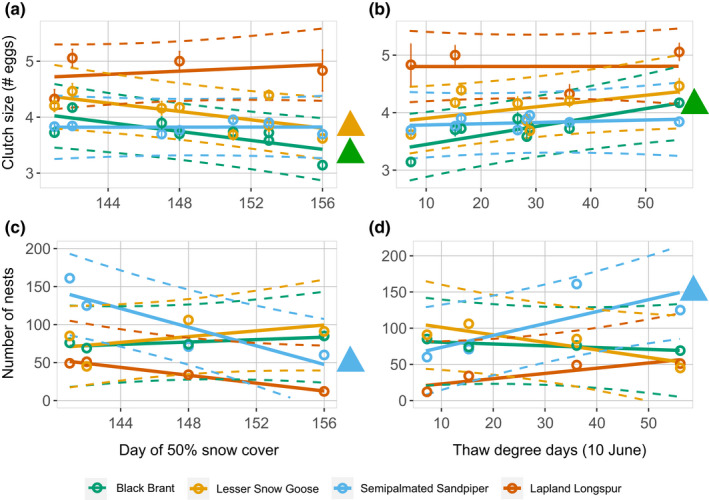
Effect of interannual differences in the date of 50% snow cover (left column) and accumulated thaw‐degree days on 10 June (right column) on the mean clutch size (a and b) and number of nests (c and d) of black brant, snow geese, semipalmated sandpipers, and Lapland longspurs at a site on the Colville River, Alaska. Clutch sizes were monitored for brant, snow geese, and semipalmated sandpipers from 2011 to 2018, and Lapland longspurs from 2015 to 2018. Nesting effort was monitored on known‐area plots for all four species from 2015 to 2018. Circles represent year‐specific values (±SE for clutch size), and solid lines represent the best‐fit least‐squares regression ±95% prediction interval (dashed lines). Species‐specific colored triangles to right of figures represent slope parameters that significantly differ from zero. See Table 3 for estimates (±95% confidence interval) of slope parameters. Ordinal date 148 is 28 May

Adjustments to the timing of reproduction in response to snow cover and spring temperature varied by species (Figure 2; Table 3). The four species arrived at the Colville River Delta earlier with advancing snowmelt (Figure 2a), but this relationship was only significant for semipalmated sandpipers (0.55 days earlier arrival for each day of advancing snowmelt; Table 3). Earlier snowmelt resulted in a reduced pre‐lay period for brant (Figure 2c; Table 3) and earlier nest initiation for all four species (Figure 2e), although the magnitude of the effect varied among species and was greatest in brant and snow geese (Table 3). Earlier snowmelt resulted in larger clutches for brant and snow geese (Figure 3a; Table 3) and increased nesting efforts by semipalmated sandpipers (Figure 3c; Table 3). In general, the effect of spring temperatures on reproductive phenology and investment was less pronounced than the effect of snow cover. Higher spring temperatures resulted in earlier nest initiation for brant and snow geese (Figure 2f; Table 3), and larger clutch sizes for brant (Figure 3b; Table 3) and higher numbers of nests for semipalmated sandpipers (Figure 3d; Table 3). Spring temperature did not influence any of the reproductive metrics for longspurs (Figures 2 and 3; Table 3).

**TABLE 3:** Response of black brant (BLBR), lesser snow geese (LSGO), semipalmated sandpipers (SESA), and Lapland longspurs (LALO) to snow cover (a; date of 50% snow cover each year) and temperature (b; accumulated thaw‐degree days from 1 January–10 June each year), 2011–2018, Colville River, Alaska

	Species			
	BLBR	LSGO	SESA	LALO
(a) Snow cover				
Site arrival	n.s.	n.s.	0.55 (0.06–1.05)*	n.s.
Pre‐lay duration	0.59 (0.04–1.14)*	n.s.	n.s.	n.s.
Nest initiation	0.84 (0.59–1.08)***	0.74 (0.50–0.98)***	0.28 (0.03–0.52)*	0.38 (0.09–0.67)*
Clutch size	−0.04 (−0.07 to −0.01)*	−0.04 (−0.07 to −0.00)*	n.s.	n.s.
Number of nests	n.s.	n.s.	−6.14 (−9.82 to −2.45)**	n.s.
(b) Temperature				
Site arrival	n.s.	n.s.	n.s.	n.s.
Pre‐lay duration	n.s.	n.s.	n.s.	n.s.
Nest initiation	−0.22 (−0.36 to −0.07)**	−0.21 (−0.36 to −0.07)**	n.s.	n.s.
Clutch size	0.02 (0.00–0.03)*	n.s.	n.s.	n.s.
Number of nests	n.s.	n.s.	1.64 (0.38–2.92)*	n.s.

Note

Values represent statistically significant slope parameters (±95% confidence intervals) from linear least‐squares regression models with date of site arrival, duration of pre‐lay period, mean date of nest initiation, mean clutch size, and number of nests as response variables. Significance levels represented by * (*p* ≤ .05), ** (*p* ≤ .01), and *** (*p* ≤ .001); n.s. indicates *p* > .05.

### 3.2 Chick growth

We modeled the growth of 166 brant goslings (99 females; 67 males) from 105 nests, 390 snow goose goslings (195 females; 195 males) from 215 nests, 250 observations of 188 semipalmated sandpiper chicks from 94 nests, and 118 observations of longspur broods from 56 nests. Excluding the intercept‐only null models, conditional *R* ^2^ values ranged from ≥.73 (snow goose) to ≥.97 (longspur) and marginal *R* ^2^ ranged from ≥.31 (snow goose) to ≥.92 (longspur) across all model sets (Table 4), indicating that the combinations of fixed and random variables in our model sets satisfactorily accounted for variation in avian growth. For all species, null models received no support (*w_i_* = 0) in multimodel comparisons, and models with only age and sex (herbivores) or age alone (insectivores) as covariates were likewise poorly supported (*w_i_* = 0; Table 4) except for longspurs, where a model fitting only age had the second‐highest model weight (*w_i_* = 0.14; Table 4) in the model set. For all species, the model‐averaged parameter estimates for juvenile age were positive (Table 5), unsurprisingly indicating the strong influence of age on body mass. Additionally, the model‐averaged parameter estimates of sex for both goose species (Table 5) indicated that male goslings weighed more than females of the same age, as expected (Ankney, 1980; Hupp et al., 2017).

**TABLE 4:** Model rankings and conditional and marginal *R* ^2^ for the relationship between resource abundance (Food), nest timing (Snow), temperature (Temp), and wind speed (Wind), and the mass of black brant and lesser snow goose goslings, semipalmated sandpiper chicks, and Lapland longspur nestlings from the Colville River, Alaska

Model^a^	*k* ^b^	ΔAIC*_c_* ^c^	*w_i_* ^d^	Conditional *R* ^2^	Marginal *R* ^2^
Black Brant					
Age + Sex + Food + Snow	7	0	0.37	.90	.71
Age + Sex + Temp + Food + Snow	8	1.84	0.15	.90	.71
Age + Sex + Wind + Food + Snow	8	2.04	0.13	.90	.71
Age + Sex + Snow	6	2.08	0.13	.90	.70
Age + Sex + Temp + Snow	7	3.27	0.07	.90	.70
Intercept only	3	133.79	0	.87	.00
Lesser Snow Goose					
Age + Sex + Temp + Wind + Snow	8	0	0.34	.74	.49
Age + Sex + Temp + Snow	7	1.12	0.2	.74	.48
Age + Sex + Temp + Wind + Food + Snow	9	1.3	0.18	.74	.49
Age +Sex + Temp + Food + Snow	8	2.8	0.08	.74	.48
Age + Sex + Temp + Wind	7	3.49	0.06	.74	.48
Intercept only	3	186.37	0	.66	.00
Semipalmated Sandpiper					
Age + Temp + Wind + Snow	8	0	0.4	.95	.78
Age + Temp + Snow	7	0.49	0.31	.95	.78
Age + Temp + Wind + Food + Snow	9	2.07	0.14	.95	.78
Age + Temp + Food + Snow	8	2.14	0.14	.95	.79
Age + Food + Snow	7	13.61	0	.95	.76
Intercept only	4	386.67	0	.74	.00
Lapland Longspur					
Age + Snow	5	0	0.17	.97	.93
Age	4	0.29	0.14	.97	.93
Age + Temp + Snow	6	0.45	0.13	.97	.93
Age + Temp	5	1.23	0.09	.97	.93
Age + Food + Snow	6	1.62	0.07	.97	.93
Intercept only	3	338.48	0	.00	.00

Note

See Table 2b for definitions of species‐specific predictor variables, and Methods for model‐set details. Except for intercept‐only models, age was included as a covariate in all models for all species, and sex in all models for brant and snow geese.

^a^ Only the top five models and intercept‐only model presented for each species.

^b^ Number of parameters. Includes terms for the intercept, residual error, and random effects.

^c^ Difference between Akaike's information criterion corrected for sample size (AIC*_c_*) and the lowest AIC*_c_* value.

^d^ Relative weight attributed to model.

**TABLE 5:** Model‐averaged predictor variables (±85% confidence interval; Arnold, 2010) from analysis of factors affecting the mass of chicks of black brant (BLBR), snow geese (LSGO), semipalmated sandpipers (SESA), and Lapland longspurs (LALO) at the Colville River, Alaska, 2012–2017

Variable	Species			
	BLBR	LSGO	SESA^a^	LALO^a^
Age	**24.611 (21.988–27.234)**	**35.983 (32.173–39.793)**	**0.571 (0.540–0.60)**	**0.899 (0.874–0.924)**
Sex^b^	**52.972 (36.812–69.132)**	**86.932 (70.628–103.236)**	n.a.	n.a.
Resource abundance	**−17.964 (−30.995 to −4.934)**	8.891 (−6.078 to 23.861)	−0.005 (−0.017 to 0.008)	0.003 (−0.010 to 0.016)
Nest timing	**−33.357 (−46.332 to −20.383)**	**−19.845 (−32.662 to −7.029)**	**−0.058 (−0.070 to −0.046)**	**−0.016 (−0.029 to −0.002)**
Temperature	−6.398 (−19.925 to 7.129)	**−107.347 (−127.507 to −87.186)**	**0.022 (0.015–0.030)**	0.008 (−0.002 to 0.018)
Wind	4.234 (−7.309 to 15.778)	**15.699 (2.935–33.077)**	**0.008 (0.001–0.016)**	−0.001 (−0.009 to 0.007)

Note

Values in bold highlight variables with confidence intervals that do not overlap 0. All predictor variables except age and sex were standardized prior to analysis; see Table 2b for species‐specific variables. The candidate models that were averaged to produce these values are presented in Table 4.

^a^ For SESA and LALO, age was log_10_‐transformed in all models to reduce heteroscedasticity.

^b^ Females are the reference level; sexes unknown for SESA and LALO.

The effect of the environmental covariates on chick growth varied by species (Table 5). The timing of nest initiation with respect to the date of 50% snow cover was the only biologically meaningful variable in common across all four species and indicated increased age‐specific body masses for chicks from nests that were initiated relatively early with respect to snowmelt (Table 5). For brant, lower resource abundance (g m^−2^ *subspathacea* biomass at 15 days of age; Table 5) was associated with increased gosling mass, and the body mass of snow goose goslings declined as thaw‐degree days increased (Table 5). For semipalmated sandpipers, higher age‐specific chick masses were associated with higher temperatures (Table 5). For longspurs, most model‐averaged parameters were small and with confidence intervals that broadly overlapped zero (Table 5), and nest timing was the only biologically meaningful predictor for this species. Higher wind speeds were associated with larger snow goose goslings and semipalmated sandpiper chicks (Table 5), but arthropod abundance did not meaningfully influence the mass of semipalmated sandpiper chicks or longspur nestlings (Table 5).

**FIGURE 4:**
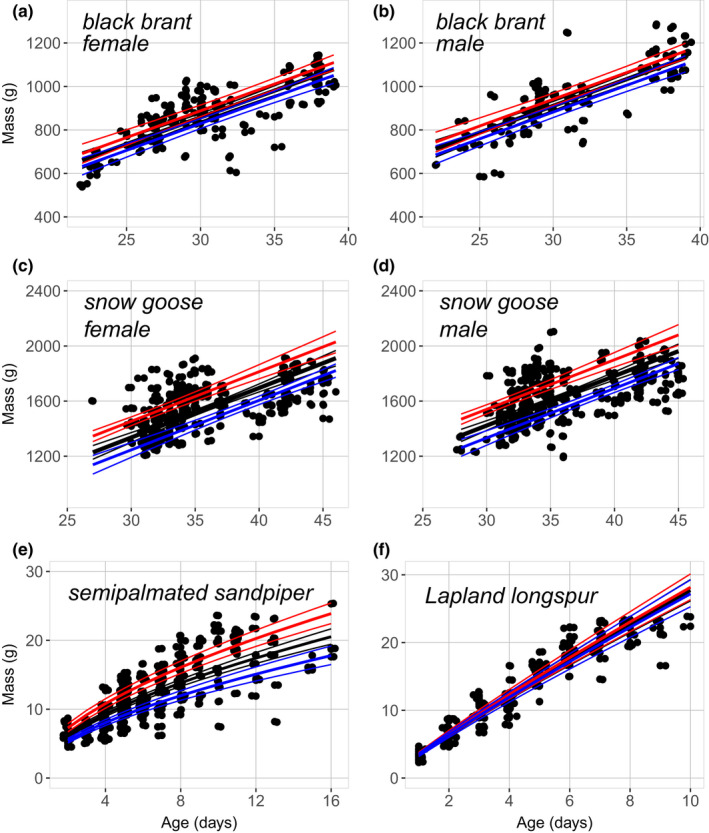
Model‐averaged predictions of chick growth of female (a) and male (b) black brant, female (c) and male (d) snow geese, semipalmated sandpipers (e), and Lapland longspurs (f) at the Colville River, Alaska. Brant and snow goose goslings measured from 2012 to 2017, semipalmated sandpiper chicks and Lapland longspur nestlings from 2015 to 2017. Measured values are represented by black circles, with overlapping values offset for clarity. Black, red, and blue lines represent body mass estimates under average, optimal, and suboptimal environmental conditions, respectively, based on variation in biologically meaningful predictor variables (see Table 5 for species‐specific variables). For all colors, heavy lines represent model‐averaged estimates, and fine lines represent the associated 95% confidence intervals

For environmental covariates that influenced chick growth (Table 5), we estimated age‐specific chick masses for each species using values of the 25^th^ and 75^th^ quartiles of these predictors to assess the effect of optimal and suboptimal (see Methods: Analysis) values of these variables on chick mass. Across the ages and sexes of chicks for which we modeled growth, the difference between optimal and suboptimal chick‐growth conditions resulted in body mass differences of 5.1%–8.5% for brant, 9.8%–15.4% for snow geese, 25.5% for semipalmated sandpipers, but only 3.4% for longspurs. Accordingly, the 95% confidence intervals on estimates derived under optimal conditions did not overlap those derived under suboptimal conditions for snow geese (Figure 4c,d) and semipalmated sandpipers (Figure 4e). The 95% confidence intervals under optimal and suboptimal conditions overlapped slightly for brant (Figure 4a,b), while those for longspurs overlapped considerably (Figure 4f).

## 4 DISCUSSION

The four species of Arctic‐breeding birds in our comparison exhibited strong variation, both within years across species and within species across years, in multiple metrics related to the timing of and investment in reproduction. This variation was also expressed during the period of chick growth, but the species‐specific responses during this phase of the breeding cycle often contrasted with those expressed during the pre‐lay and nesting phases. Our prediction that spring temperatures and snow cover would have a greater effect on the timing of nest initiation and clutch size in geese compared to semipalmated sandpipers and longspurs was generally supported and may reflect the differing role that endogenous reserves play among the taxa during reproduction (see below). Our prediction that arthropod abundance would have relatively little effect on the growth of altricial longspur nestlings was also supported, but we found no evidence that resource abundance influenced the growth of semipalmated sandpiper chicks or snow goose goslings. Furthermore, the effects of *subspathacea* biomass on the growth of brant goslings were opposite of our predictions, emphasizing the role of factors other than resource abundance in juvenile growth.

### 4.1 Annual adjustments to reproductive phenology and investment

Of the four species in our comparison, only semipalmated sandpipers meaningfully adjusted their arrival date in response to environmental conditions (snow cover), likely a reflection of the species’ dependence on exogenous reserves derived from arthropod prey that only become available as snowmelts and temperatures warm (Holmes & Pitelka, 1968; MacLean & Pitelka, 1971). In contrast, snow geese and longspurs consistently arrived the earliest at our study site each spring. Snow geese employ their robust bill and specialized foraging techniques to access nutritious subsurface roots and shoots (Iacobelli & Jefferies, 1991), while longspurs can subsist on seeds (Custer & Pitelka, 1978) prior to the emergence of arthropods. Brant are relatively dependent on food resources that emerge only as temperatures warm and melting snow exposes appropriate foraging substrates (Lewis et al., 2020), but unlike semipalmated sandpipers, brant also carry significant endogenous reserves that can serve as buffers when food resources are inaccessible (Hupp et al., 2018).

Subsequent phases of the reproductive cycle of the species in our comparison further reflected the species’ positions on the endogenous–exogenous continuum. Geese are capable of carrying comparatively larger endogenous reserves to the Arctic that they can then invest in egg production and incubation (Klaassen et al., 2006). Conversely, shorebirds and passerines derive virtually all egg nutrients and reserves for self‐maintenance after their arrival to the breeding area. All species advanced nest initiation with earlier snowmelt, but the effect was strongest for geese, which also advanced nest initiation in response to warmer spring temperatures. In years when Arctic phenology is advanced, geese can use their reserves to begin egg development in late migration or shortly after their arrival on the nesting area so as to initiate nests early and better time the hatch of offspring with peak nutrient availability (Klaassen et al., 2006; Nolet et al., 2020). Compared to the large‐bodied goose species that can rely on endogenous reserves, the pre‐lay periods for semipalmated sandpipers and longspurs were relatively long, reflecting the fact that individuals of both species must first forage to acquire the necessary exogenous resources prior to producing eggs. Relative to snow geese, brant invest more endogenous nutrients into eggs, and in years when spring is advanced are more likely to initiate follicle development during migration (Hupp et al., 2018). The shorter pre‐lay durations in brant during years of earlier snowmelt likely reflected the species’ tendency to initiate follicle development prior to arrival on the nesting area. Follicle development in snow geese breeding on the Colville River Delta mainly occurs after arrival in the Arctic (Hupp et al., 2018), and their pre‐lay interval is less variable relative to environmental conditions.

Although endogenous investment in eggs gives geese an advantage in advancing egg development and nest initiation when Arctic phenology is advanced, female geese may use reserves for self‐maintenance at the expense of reproductive investment in years when snow and cold temperatures persist (Barry, 1962; Raveling, 1978; Reed et al., 2004). Because brant invest relatively more endogenous reserves into eggs (Hupp et al., 2018) than the other species we studied, they are more likely to reduce clutch sizes in colder springs or when late snowmelt delays nest initiation. In contrast, phylogenetic constraints (MacLean, 1972) likely preclude semipalmated sandpipers from varying clutch sizes (Sandercock et al., 1999), although previous research indicates that shorebirds can regulate egg size within clutches in response to seasonal variation (Martin et al., 2018; Sandercock et al., 1999). Longspurs exhibited the greatest variation in clutch size during our study (1–7 eggs), but this variation was not related to environmental conditions. Such variation may instead reflect the influence of other factors (e.g., female age or breeding experience; Sæther, 1990; Stearns, 1976) that we did not measure.

In addition to adjusting investment in clutches, Arctic‐breeding birds may forego breeding altogether in response to extreme environmental conditions (Ganter & Boyd, 2000; Schmidt et al., 2019), a response believed to reflect a trade‐off between current reproductive investment and future survival (Linden & Møller, 1989; Roff, 2002). The nesting effort of semipalmated sandpipers was greatest in years with warm temperatures and early snowmelt, but the other three species did not meaningfully moderate nesting efforts in response to these variables. This variation again suggests the likely role of exogenous reserves in modulating the reproductive output of semipalmated sandpipers. Shorebirds lay clutches that constitute a relatively high proportion of their body mass (Rahn et al., 1975; Ricklefs, 1984), and semipalmated sandpiper females at our study site may have been unable to acquire sufficient arthropod resources to initiate nests in cold springs with extensive snow cover. In contrast, brant and snow geese could rely on endogenous reserves, and longspurs could exploit seed resources in lieu of arthropods, to ensure nesting opportunities.

### 4.2 Variation in chick growth

Interannual adjustments in the timing of and investment in breeding may reflect adaptive responses to prevailing environmental conditions, but nests must hatch and chicks must grow and survive in order for such adjustments to be propagated in an evolutionary context (see Charmantier & Gienapp, 2014). The rate of chick growth provides insights into fitness‐related variables (e.g., survival, recruitment, lifetime reproductive output) that are otherwise extremely difficult to measure in most bird species. Curiously, resource abundance was not a meaningful predictor of chick mass of either insectivore species in our study. Obviously, food abundance directly affects chick growth, which suggests that either we did not measure arthropod abundance in a way that reflected real abundance or that the range of abundances that we measured at our site did not limit growth. For the former supposition, this same sampling protocol has been successfully employed by others (see Kwon et al., 2019 for overview), and Saalfeld et al. (2019) specifically determined that this sampling technique described variations in arthropod abundance that predicted the chick mass of two shorebird species (dunlin *Calidris alpina* and pectoral sandpiper *Calidris melanotos*) that are closely related to semipalmated sandpipers. Thus, it is more likely that arthropod abundances did not limit chick growth during our period of study. Despite measuring a nearly 300‐fold variation in arthropod abundance (1.5–427.3 mg 3‐day^−1^ sample) during periods of chick growth from 2015 to 2017, our measurements did not apparently reflect conditions that affected the growth of insectivore chicks. For semipalmated sandpipers, higher temperatures in the 3‐day period prior to recapture, however, were associated with larger same‐age chicks. In general, arthropod abundance was low at lower temperatures and increased at temperatures >5°C, but, importantly, we also documented periods of high temperature that coincided with low arthropod abundance during which we nonetheless observed high age‐specific chick masses. This suggests a thermogenic trade‐off for semipalmated sandpiper chicks at higher temperatures wherein the high cost of thermogenesis (Bakken et al., 2002; Schekkerman et al., 2003) may be minimized and growth maximized (McKinnon et al., 2013), permitting rapid chick growth even during periods of relatively low food abundance (but see Saalfeld et al., 2021).

Resource abundance was a meaningful predictor of the growth of only brant, but the negative effect was counterintuitive. Previous research has demonstrated an inverse relationship between forage biomass and forage quality (i.e., nitrogen content) in graminoids like *subspathacea* (Doiron et al., 2014; Flint & Meixell, 2021; Lameris et al., 2017), a relationship which may account for this finding. There is a strong positive relationship between nitrogen content and demographic variables like gosling growth and survival (Doiron et al., 2015; Manseau & Gauthier, 1993; Person et al., 2003; Sedinger & Raveling, 1986), and in our study, it may be that periods of high *subspathacea* biomass had correspondingly low values for nitrogen content. Thus, the association between higher age‐specific body mass of brant goslings and lower forage biomass may reflect aspects of forage quality that we were unable to measure.

In contrast, we did not find that higher *subspathacea* biomass negatively affected the growth of snow goose goslings. Research from other sites in the species’ breeding range, however, has demonstrated that the growth of snow goose goslings can be limited by resource abundance (Lepage et al., 1998; Lindholm et al., 1994). These studies were conducted at a breeding site with degraded grazing lawns and low‐quality food compared to that on the Colville River (Hupp et al., 2017), emphasizing how spatial variation in ecological factors—in this case, food quality and abundance—can differentially affect the demographic response of the same populations (Sedinger et al., 2001). At the Colville River, the larger snow goose goslings may be able to accommodate lower quality *subspathacea* compared to smaller brant goslings due to a greater intake and processing capacity (Lesage & Gauthier, 1997; Manseau & Gauthier, 1993; Richman et al., 2015). Further, in our study, larger snow goose goslings were associated with cooler temperatures. This result may again indirectly reflect aspects of food quality (Dickey et al., 2008) rather than thermal constraints on growth per se (but see Fortin et al., 2000) because *subspathacea* responds to warm temperatures with increased vegetative growth but decreased nitrogen content (Doiron et al., 2014; Flint & Meixell, 2021; Lameris et al., 2017). Thus, although we do not fully understand the mechanisms acting at the Colville River, our results demonstrates that higher temperatures during chick rearing can differentially affect growth rates of avian herbivores versus insectivores.

Of note, the environmental covariates that we assessed in our analysis did not strongly affect the mass of longspur nestlings, the only altricial species in this comparison. Only the timing of nest initiation was a biologically meaningful predictor in our analysis (Table 5), but the effect of this variable on nestling growth was trivial (Figure 4f) compared to those affecting the other three species (Figure 4a–e). Previous research at a nearby site in Arctic Alaska similarly documented seasonal declines in the growth of longspur nestlings, but also negative effects of low arthropod abundance and cold temperatures (Pérez et al., 2016). At our study site, however, other factors were apparently more important in modulating the growth of longspurs. It may be that aspects of parental quality that we did not measure (e.g., nest‐site selection [Martin et al., 2000, Lloyd & Martin, 2004], chick provisioning [Davies, 1986; Limmer & Becker, 2009]) buffered deleterious effects of temperature and resource abundance that affected the chicks of precocial species at our site.

An environmental variable that received wide support across our comparisons was the timing of nest initiation with respect to snow cover. For all four species, chicks from nests that were initiated before or near the annual date of 50% snow cover were larger than same‐age chicks from nests that were initiated relatively later. The positive effect of early initiation on chick growth has been documented in other studies of Arctic‐breeding birds (Cooch et al., 1991; Ruthrauff & McCaffery, 2005; Sedinger & Flint, 1991), and our study indicates that early nest initiation rather than resource abundance is a more important factor in regulating chick growth. This suggests the role of potential factors such as parental quality (Clutton‐Brock, 1984; Forslund & Pärt, 1995) or carry‐over effects (Harrison et al., 2011) that we could not measure.

## 5 CONCLUSION

Notably, the four species in our study share the overarching life‐history trait of being migratory animals. Previous research has suggested that migratory species are more vulnerable to climate change due to the potential decoupling of relevant seasonal cues across a species’ range (Both et al., 2010; Møller et al., 2008; Robinson et al., 2009). We documented interannual responses among these four migratory species, however, that demonstrated a high degree of interspecific response to shared stimuli among diverse taxa. The species at our study site use two flyways (Pacific [brant and snow goose] and Central [snow goose, semipalmated sandpiper, and longspur] Americas flyways) across a mix of marine (brant and semipalmated sandpiper), seasonal wetland (snow goose and semipalmated sandpiper), and agriculture/prairie landscapes (snow goose and longspur) to migrate to Arctic breeding grounds. Despite the use of varied migratory routes and habitats, these species nonetheless adjusted their timing of breeding in ways that tracked spring environmental conditions at the breeding site. Indeed, long‐term information for these and other species at this site shows a general advancement of arrival dates in concert with warming spring conditions (Ward et al., 2016), patterns noted more broadly in other studies (Jonzén et al., 2006; Thorup et al., 2007; Van Buskirk et al., 2009). Migratory birds exhibit life histories that are predicated on exploiting diverse, ephemeral landscapes (Greenberg & Marra, 2005; Newton, 2008), and employ flexible physiologies that permit the rapid hypertrophy and subsequent atrophy of respiratory, digestive, and circulatory systems (Piersma & van Gils, 2011). Such behavioral and physiological adjustments enable large‐scale movements and undoubtedly also serve as buffers in a changing world. So although migratory species may theoretically be predisposed to a decoupling of seasonal cues across their large ranges, these four species responded predictably to prevailing environmental conditions by adjusting their reproductive timing and investment. Migratory birds, by virtue of their intrinsic life histories, may thus accommodate the effects of a warming Arctic better than previously appreciated.

More specifically for these four species, life‐history traits that afford flexible responses to variable environmental conditions are favored in highly seasonal and unpredictable environments like the Arctic. Traits that in turn promote evolutionary changes in a population are further expected to be subject to strong selection pressure under climate‐warming scenarios (Berteaux et al., 2004; Hoffmann & Sgrò, 2011; Williams et al., 2008). Temperatures across all seasons are projected to increase on Alaska's Arctic Coastal Plain due to climate change (IPCC, 2021), and increases in warming have already led to long‐term advances in snowmelt and longer snow‐free seasons in Arctic Alaska (Cox et al., 2017; Hinzman et al., 2005; Stone et al., 2002). Brant and snow geese generally responded more flexibly to variation in temperature and snowmelt during the pre‐lay and nesting periods than did semipalmated sandpipers and longspurs. In contrast, we detected potentially deleterious effects of increased temperature on brant and snow goose goslings, while semipalmated sandpiper chicks responded favorably to warmer conditions. Thus, brant and snow geese may possess traits that are beneficial during one phase of the reproductive cycle (e.g., relative flexibility along the endogenous–exogenous spectrum) and others which may be detrimental at another phase (e.g., temperature‐mediated sensitivity to food quality during juvenile growth). For the Arctic‐breeding birds in our study, these contrasting responses underscore the importance of assessing the effects of climate variability across multiple phases of the reproductive cycle (Nolet et al., 2020).

## CONFLICT OF INTEREST

We have no competing interests.

## AUTHOR CONTRIBUTION

**Daniel Ruthrauff:** Conceptualization (lead); Data curation (equal); Formal analysis (lead); Funding acquisition (equal); Project administration (equal); Writing – original draft (lead); Writing – review & editing (lead). **Vijay P. Patil:** Conceptualization (equal); Data curation (equal); Formal analysis (supporting); Investigation (equal); Writing – review & editing (supporting). **Jerry W. Hupp:** Conceptualization (equal); Funding acquisition (equal); Investigation (equal); Methodology (equal); Project administration (equal); Writing – review & editing (supporting). **David H. Ward:** Conceptualization (equal); Funding acquisition (equal); Investigation (equal); Project administration (equal); Writing – review & editing (supporting).

## Data Availability

Data collected in support of this manuscript are publicly available at the U.S. Geological Survey Alaska Science Center's data archive: https://doi.org/10.5066/F72J692N, https://doi.org/10.5066/F7M907KT, https://doi.org/10.5066/P9BJBRTO, https://doi.org/10.5066/P9UE2Q73, https://doi.org/10.5066/P9AMSIEJ, and https://doi.org/10.5066/P9CDID03.
